# Distinguishing Fibroepithelial Lesions Requires Clinical, Imaging, and Pathology Correlation

**DOI:** 10.7759/cureus.47673

**Published:** 2023-10-25

**Authors:** Megan Bradley, Brittany Miles, Peter Young, Jing He, Quan D Nguyen

**Affiliations:** 1 John Sealy School of Medicine, University of Texas Medical Branch at Galveston, Galveston, USA; 2 Radiology, University of Texas Medical Branch at Galveston, Galveston, USA; 3 Pathology, University of Texas Medical Branch at Galveston, Galveston, USA; 4 Radiology, Baylor College of Medicine, Houston, USA

**Keywords:** management of phyllodes tumor, ultrasound, mammography, breast fibroadenoma, phyllodes tumors

## Abstract

Phyllodes tumor (PT) is a rare tumor that can present as benign, borderline, or malignant. These tumors arise from the breast stroma, similar to fibroadenomas. Phyllodes tumors and fibroadenomas often have overlapping features in both radiological imaging and pathologic analysis. As a result, these two lesions are often difficult to differentiate and require the correlation of multiple modalities, including clinical context, radiologic imaging, and histological evaluation. This article presents a case of a borderline phyllodes tumor in a 51-year-old female, with the inclusion of its radiologic and pathologic images and performed treatment. The goal of this article is to provide a review of the clinical presentation, diagnostic imaging and pathology features, treatment, and management of a phyllodes tumor and compare and contrast this against the more common fibroadenomas, in order to provide aid for differentiating these two breast lesions.

## Introduction

Phyllodes tumor (PT) is a rare fibroepithelial lesion that accounts for 0.3-0.9% of all breast tumors [1]. This mass typically presents in middle-aged women, peaking at around 40-50 years of age [2], which is approximately 15-20 later than the peak age of fibroadenomas [2]. While there have been a few reports of PTs in men, it is extremely rare and is therefore more prevalent in women [3].

PT typically presents as a painless, rapidly growing mass [1], ranging in size from 2 to 40 cm, with a mean of 7 cm [3]. Associated symptoms, such as nipple retraction or ulceration, are uncommon on presentation for this tumor type [2]. While these tumors often present with axillary lymphadenopathy, metastasis to the axillary lymph nodes is rare [2].

The World Health Organization (WHO) classifies PTs as either benign, borderline, or malignant, with benign being the most common, representing 60-75% of PT cases [4]. These categorical assignments are dependent on histological characteristics including tumor margins, mitotic count, and stromal cellularity, overgrowth, and atypia [1]. Even with the development of these histological characteristics, there is no clearly defined cutoff within these parameters for the diagnosis of a PT [4]. Since the diagnosis of PTs heavily relies on histology and the pathologist’s expertise, the diagnosis between benign PTs and fibroadenomas remains challenging [2]. In our case, we present a case of a borderline PT in a 51-year-old African-American woman.

## Case presentation

A 51-year-old woman was found to have a left breast mass on a routine mammogram screening. On physical examination, a 4 x 4 cm mass was seen at the 8 o’clock position without associated edema, dimpling, rashes, or nipple discharge. Furthermore, no axillary or supraclavicular lymphadenopathy was palpated on physical examination.

**Figure 1:**
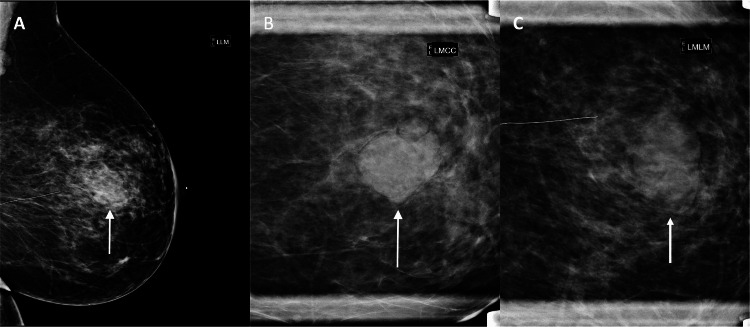
Mammography of the left breast demonstrates a 3.8 cm, equal-density, oval mass with indistinct margins located at the lower inner quadrant, anterior depth, 4 cm from the nipple This correlates with the palpable mass reported by the patient. (A): Lateromedial (LM) view; (B): Magnified craniocaudal (CC) view; (C): Magnified LM view

**Figure 2:**
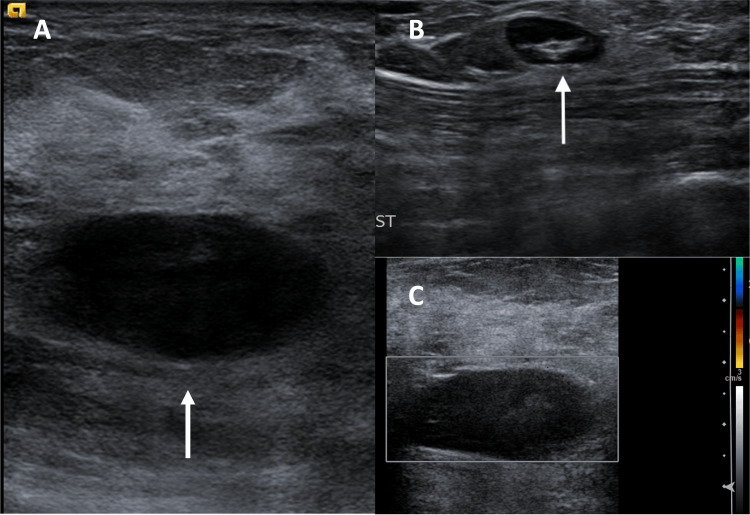
(A) Transverse ultrasound of the left breast demonstrates a 3.5 x 1.5 x 3.0 cm hypoechoic oval mass at the 8 o’clock position, 4 cm from the nipple. (B) Ultrasound of the left axilla demonstrates a 1.0 cm lymph node with mild cortical thickening. The axillary lymph node was determined to be benign with no identifiable malignancy according to pathology. (C) Transverse ultrasound with color Doppler showing no color vascular flow.

Mammography demonstrated an equal-density, oval-shaped mass with indistinct margins in the lower inner quadrant (Figure 1). The ultrasound showed a 3.5 x 1.5 x 3.0 cm solid, hypoechoic mass, and an axillary lymph node with cortical thickening (Figure 2). The lesion was categorized as Breast Imaging Reporting and Data System (BI-RADS) category 4B, and an ultrasound-guided biopsy was recommended.

**Figure 3:**
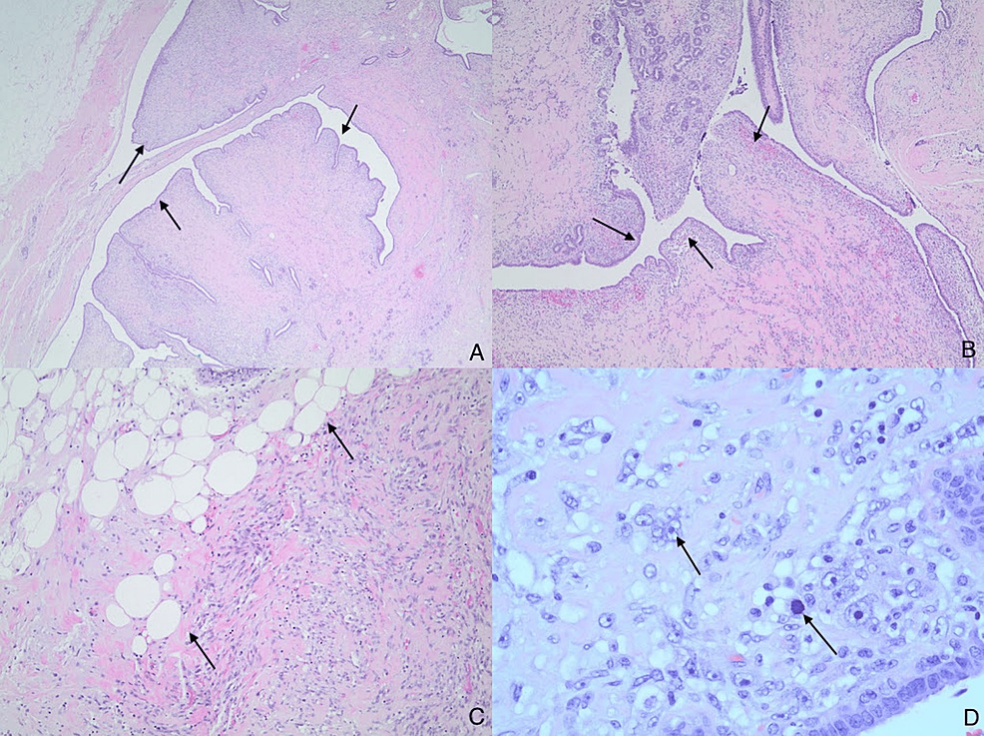
Histological findings of lumpectomy of the borderline phyllodes tumor (A): Lumpectomy of the left breast shows a leaf-like (phyllodal) pattern by an exaggerated intracanalicular pattern (magnification 20x). (B): Subepithelial condensation with increased stromal cellularity adjacent to the epithelium (magnification 40x). (C): Fibroepithelial lesion invading adipose tissue (magnification 100x). (D) Stromal cells demonstrate cytological atypia with nuclear pleomorphism, vesicular nuclei, irregular nuclear contours, prominent nucleoli, and mitotic figures (magnification 400x).

Biopsy results showed a fibroepithelial neoplasm, favoring a PT. Surgical excision was recommended, and the patient underwent a lumpectomy with sentinel lymph node biopsies (Figure 3). The final pathology report details a borderline PT and benign sentinel lymph nodes. The tumor was identified as stage 1 (T1N0M0), and no adjuvant therapy was performed. The patient has since been lost to follow-up.

## Discussion

While most breast carcinomas involve the ducts or lobules, PTs involve the breast stroma, similar to fibroadenomas [2]. As a result, differentiating between these two breast masses is often difficult, requiring multiple modalities in addition to clinical context. On mammography, fibroadenomas commonly present as an oval mass with well-circumscribed margins, whereas PTs are more likely to present as an oval mass with irregular margins; however, these features overlap [5]. Ultrasound findings showing an anechoic oval mass is diagnostic of a cystic mass while a hypoechoic oval mass is characteristic of a solid mass. PTs can present with increased heterogeneity and vascularity on ultrasound compared to fibroadenomas, but these characteristics overlap [5]. The ultrasound in this case was difficult to interpret, as the mass could be anechoic or hypoechoic, with no signs of increased heterogeneity or vascularity, providing no additional diagnostic certainty in distinguishing among a PT, fibroadenoma, or cyst.

Cysts are classified as BI-RADS 2, indicating a benign lesion and requiring no further workup. Fibroadenomas are typically classified as BI-RADS 3, indicating a likely benign lesion and requiring a follow-up ultrasound within six months. The lesion in this patient's case was determined to be BI-RADS 4B, indicating a moderate suspicion for malignancy and required further workup with biopsy. This category assignment was likely due to both the suspicious imaging findings and the clinical context of a large mass in a 51-year-old female. Clinical context, as in this patient's case, is often the only diagnostic signal that this lesion is a possible PT as opposed to fibroadenoma since these lesions can present similarly on radiologic imaging modalities [6].

Fine-needle aspiration cytology (FNAC) is unreliable in discerning between benign PTs and fibroadenomas [7], with a sensitivity of only 40% for diagnosing PTs [8]. Core needle biopsies (CNBs) are more accurate in establishing a diagnosis, with an improved sensitivity of 63% [8]. The overall sensitivity for distinguishing between PTs and fibroadenomas is improved to 76% when cytohistological results are combined with radiologic imaging modalities [8]. PTs demonstrate a histological pattern of leaf-like architecture, but this pattern can also be seen in fibroadenomas and is therefore not diagnostic [4]. Other characteristics, such as mitotic index, stromal overgrowth, and stromal cellularity, can be used to help further distinguish these lesions, but there is still morphological overlap [4]. As a result, the diagnosis relies upon the pathologist’s expertise. 

If a patient is diagnosed with either a benign or a borderline PT, imaging for tumor staging does not need to be performed [9]. If a malignant PT is identified, patients should undergo a staging CT scan of the chest since the lung is the most common site for metastasis [9]. Surgical excision is the standard of care for all PTs [9]. On the contrary, fibroadenomas are usually managed conservatively, though surgical excision may be required if the tumor is rapidly growing or if the patient is symptomatic [10]. All three types of PTs have a high rate of recurrence (17% in benign and 27% in malignant) [4], and the probability of recurrence is greatest in the first two years following surgical excision [9]. For surveillance, patients with borderline PTs should have an ultrasound of the ipsilateral breast performed every six months during this time frame [9]. For patients with malignant PTs, regular visits with imaging, including CT scans of the chest, later transitioning to chest X-rays, and yearly ultrasounds of the breast should be performed for three years [9].

## Conclusions

The diagnosis of a PT should be considered in all middle-aged women who present with a rapidly growing, palpable breast mass with limited associated symptoms. While PTs, especially benign PTs, present similarly to fibroadenomas, a combination of presentation, imaging modalities, and biopsy may be used to enhance diagnostic certainty for differentiating these tumors. This case brings further attention to the presentation, diagnostic evaluation, and appropriate treatment plan for a PT that is challenging to differentiate from the more common fibroadenoma.
